# Machine learning–based personalized prediction of developmental dysplasia of the hip using risk factors and clinical examination

**DOI:** 10.3389/fdgth.2026.1912106

**Published:** 2026-10-14

**Authors:** Shuang-shuang Zhang, Gianfilippo Caggiari, Xiao-yi Chen, Bing-xuan Huang, Han-sheng Deng, Na Xu, Wei Shi

**Affiliations:** 1 Department of Medical Ultrasound, Jinhua Municipal Central Hospital, Zhejiang, China; 2 Department of Medicine, Surgery and Pharmacy, University of Sassari, Sassari, Italy; 3 Orthopedic and Traumatology Unit, Azienda Ospedaliero-Universitaria di Sassari, Sassari, Italy; 4 Department of Medical Ultrasound, The Central Hospital of Wuhan, Tongji Medical College, Huazhong University of Science and Technology, Hubei, China; 5 Department of Medical Ultrasonics, Affiliated Shenzhen Children's Hospital of Shantou University Medical College, Shenzhen, China; 6 Department of Orthopedics, Affiliated Shenzhen Children's Hospital of Shantou University Medical College, Shenzhen, China; 7 Department of Medical Ultrasonics, Shenzhen Children's Hospital & Shenzhen University Medical School, Shenzhen, China

**Keywords:** developmental dysplasia of the hip, extreme gradient boosting, light gradient boosting machine, logistic regression, machine learning

## Abstract

**Background:**

Machine learning–based disease prediction models can assist with disease diagnosis and early detection. This study aimed to identify the risk factors for developmental dysplasia of the hip (DDH) and develop multiple machine learning–based models to support screening and risk stratification for DDH.

**Methods:**

A retrospective analysis of 12,105 infants screened for DDH between January 2020 and December 2022 at Shenzhen Children’s Hospital was performed to collect information on physical examination and risk factors. After univariate and multivariate screening, age-stratified sampling was performed on 11,898 DDH-negative patients to match the age distribution of 207 DDH-positive patients, thereby reducing potential bias. These balanced cohorts were randomly split into training and test sets (at an 8:2 ratio) under both 1:1 and 1:2 positive-to-negative ratios. Predictive modeling was based on random forest, support vector machine, logistic regression, k-nearest neighbor, light gradient boosting machine, and extreme gradient boosting (XGBoost).

**Results:**

Twelve variables were included in the model. The XGBoost model trained on 1:1 data was chosen as the best model [area under the curve (AUC): 0.883, accuracy: 0.807, precision: 0.744, F1 score: 0.800, recall (sensitivity): 0.865, and specificity: 0.761]. External validation using data from Jinhua Municipal Central Hospital provided preliminary evidence of transferability [AUC: 0.909, accuracy: 0.838, precision: 0.829, F1 score: 0.840, recall (sensitivity): 0.850, and specificity: 0.825]. Skin pattern asymmetry, restriction of hip abduction, swaddling, breech presentation, and female sex were the top five most important predictive variables.

**Conclusions:**

The XGBoost model can be used to support early clinical screening and risk stratification for DDH, helping clinicians identify infants who should undergo hip ultrasonography.

## 1 Introduction

Developmental dysplasia of the hip (DDH), which encompasses acetabular immaturity, dysplasia, and dislocation, is a common bone and joint disorder in infants. The prevalence of DDH varies greatly depending on region, race (ranging from 0.1% in Africans to 7.6% in Native Americans), and diagnostic criteria (1, 2). Most patients who receive early and effective treatment can regain normal hip structure and function; however, cases of delayed diagnosis and treatment in patients are more complex and may lead to varying degrees of disability (3). There is no uniform standard for ultrasound screening of the target population (4) because a universal ultrasound screening standard requires extensive medical resources. However, selective ultrasound screening may delay the treatment of some children with DDH. Therefore, predicting the risk for DDH, selecting the target population, and improving the efficiency of DDH diagnosis are the focus areas of current research.

An epidemiological investigation of DDH revealed that many factors influenced DDH, highlighting that their relationships are intricate and complex. With the advancement of technology, disease prediction models based on machine learning (ML) can assist with the diagnosis and early detection of diseases that have complex influencing factors (5, 6), with significantly better predictive efficacy than traditional statistical methods (7, 8).

This study developed and validated a machine learning model to support screening and risk stratification for DDH in infants using a large dataset, with the goal of improving the early detection rate of DDH. In addition, we aimed to identify the risk factors for DDH and develop multiple ML-based models to support screening and risk stratification for DDH, so as to improve the early detection rate and facilitate timely treatment. The correlation between risk factors and DDH development was elucidated with Shapley additive explanation (SHAP), a method that quantifies how much each variable pushes a model's probability upward or downward, thereby helping clinicians interpret the model's output.

## 2 Methods

### 2.1 Study participants

We retrospectively analyzed and collected information on 12,105 (207 patients with DDH, 1.7%) infants who underwent DDH screening at Shenzhen Children's Hospital between January 2020 and December 2022 to establish the training and testing sets of the model. In addition, we retrospectively analyzed and collected information on 80 (50% positive) infants who underwent DDH screening at the ultrasound department of Jinhua Municipal Central Hospital between 1 January 2024 and 31 December 2024 to create the validation set for external validation of the model. The inclusion criteria are given as follows: ages between 14 days and 6 months, a history of at least one hip ultrasound and physical examination, and no neurological abnormalities on physical examination. The exclusion criteria are given as follows: intervention before the first visit, presence of syndromes, and comorbidity with other hip disorders (pathological dislocation, paralytic dislocation, spastic dislocation of the hip, teratoid dislocation, and a history of hip infection or hip arthritis). The screening procedure for participant recruitment at Shenzhen Children's Hospital is shown in Figure 1.

**Figure 1:**
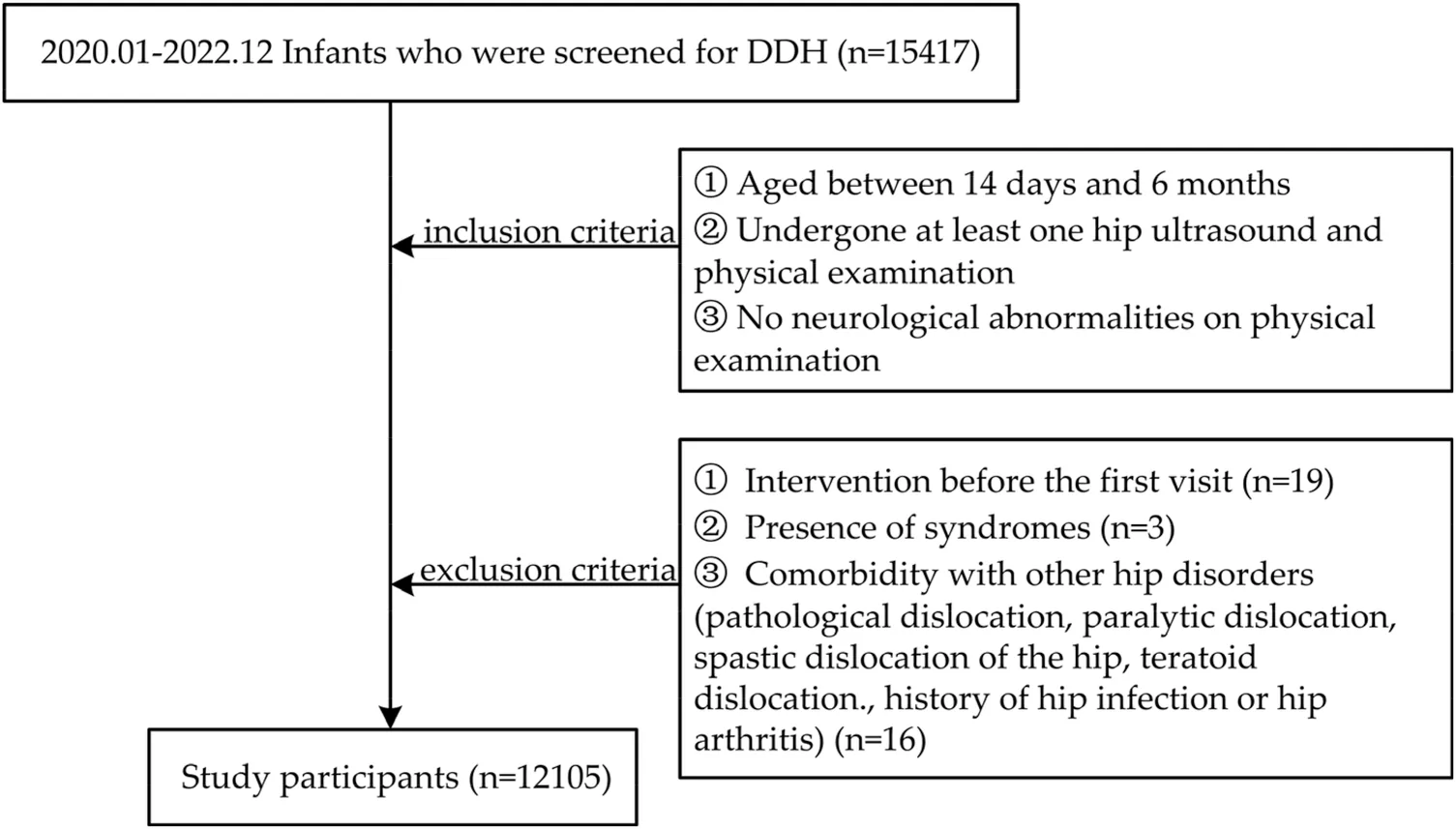
A flowchart showing the procedure for recruiting participants according to the inclusion and exclusion criteria.

This study was conducted in accordance with the Declaration of Helsinki, was approved by the Ethics Committee of Shenzhen Children's Hospital (approval no. 2022078) and the Ethics Committee of Jinhua Municipal Central Hospital (approval no. 2025–94), and was exempt from written informed consent.

### 2.2 Data collection and classification

All patients underwent a Graf method–based ultrasound examination to confirm the diagnosis of DDH (including types I–IV). A clinical examination was performed by an experienced orthopedic surgeon (>20 years of experience) to determine whether the patients met the standardized diagnostic criteria for DDH, including restriction of hip abduction (≥20°) and leg length discrepancy (Galeazzi's sign). In addition, the presence of a stooped neck, congenital foot deformities (clubfoot, calcaneal exostosis, etc.), and an asymmetry of thigh and groin skin folds was recorded. A standardized questionnaire, administered electronically by uniformly trained investigators, was used to collect information on basic details and risk factors, including age (in months), birth weight, sex, birth in winter (December–February), premature vaginal birth or cesarean section, multiple births, number of deliveries, breech presentation or breech delivery in late pregnancy (≥28 weeks of gestation), a history of oligohydramnios (≥28 weeks of gestation, ultrasound suggestive of deepest vertical pocket ≤2 or amniotic fluid index ≤5), swaddling, and frog leg position. A first-degree family history of DDH was defined as a diagnosis reported in one or both biological parents; however, no information on their affected siblings was collected.

### 2.3 Statistical analysis

SPSS (version 26.0; IBM) software was used for statistical analysis. Numerous factors influence DDH, and we selected only 17 variables for inclusion into the model because of the small DDH-positive sample size. One-way analyses of categorical and continuous variables (normally distributed) were performed using chi-square tests and independent sample t-tests, respectively, to exclude indicators with *p* > 0.1. A multifactor logistic regression (LR) analysis was performed to screen independent variables, and variables with *p* < 0.05 were used as candidates for building ML models.

This statistical prescreening method was chosen for two reasons: (i) to constrain model complexity and reduce the risk of overfitting given the relatively small number of patients with DDH and (ii) to anchor the models to clinically established predictors with interpretable odds ratios familiar to clinicians.

### 2.4 ML model building

In this study, the ML model was built using Python 3.9.18; the libraries used include pandas 1.3.4, NumPy 1.24.3, SciPy 1.11.3, scikit-learn 1.6.1, light gradient boosting machine (LightGBM) 3.3.5, extreme gradient boosting (XGBoost) 1.7.5, and SHAP 0.41.0. Before machine learning model training, we conducted a clinical-driven variable prescreening using the full source cohort of 12,105 infants from Shenzhen Children's Hospital. This prescreening step, including univariate and multivariate logistic regression, aimed to identify clinically plausible predictors and reduce model complexity given the limited number of patients with DDH. The resulting set of 12 candidate predictors was fully fixed prior to any sampling or dataset partitioning.

To address potential prediction bias driven by age confounding, we performed age-stratified undersampling among the 11,898 patients without DDH to match the age distribution of the 207 patients with DDH. As shown in Table 1, DDH prevalence decreased markedly with an increase in the age of the infants. This matching strategy was implemented to remove the strong confounding effect of age, ensuring that the models learned true associations between risk factors and DDH status rather than merely capturing age differences; direct use of the raw imbalanced cohort would allow age to dominate model predictions. Based on two positive-to-negative ratios (1:1 and 1:2), we generated two balanced cohorts. Each balanced cohort was randomly split into a training set and a held-out test set at an 8:2 ratio with a fixed random seed (‘random_state’ = 42). All hyperparameter optimization, 10-fold cross-validation, and model fitting were performed exclusively on the training set. The held-out test set was blinded throughout model development and was used only for final model performance evaluation. np.random.seed(10) was set before hyperparameter optimization, and a fixed seed was used for all models to ensure exact reproducibility. Notably, the 10-fold cross-validation (KFold, n_splits = 10, shuffle = True, random_state = 123) was used as an internal validation mechanism to evaluate the model's overfitting risk and stability. Six ML algorithms with excellent prediction capabilities and high robustness—random forest (RF), support vector machine (SVC), LR, k-nearest neighbor (KNN), LightGBM, and XGBoost (9, 10)—were selected to build the prediction model. A grid search (GridSearchCV, cv = 3, scoring = ‘roc_auc’) was performed to select the best parameters for each model; the complete search grids and the final optimal parameters for both the 1:1 and 1:2 cohorts are listed in Supplementary Table S1. The receiver operating characteristic (ROC) curve, area under the curve (AUC), accuracy, precision, F1 score, recall (sensitivity), and specificity were used to evaluate and compare the performance of each model. Recall (sensitivity) measured the proportion of actual patients with DDH who were correctly identified by the model, indicating its ability to avoid false-negative results. The 90% CIs of the AUC were estimated by bootstrap resampling (1,000 iterations). In addition, we will externally validate the model with external data obtained at Jinhua Municipal Central Hospital. SHAP is an ML model visualization tool that can interpret the output of any ML model by calculating how much each feature contributes to the model output and can be used to interpret the model at both the local and global levels (11, 12). The best-performing model was interpreted using SHAP (TreeExplainer for XGBoost; KernelExplainer for SVC).

**Table 1:** General characteristics of the study participants at baseline.

Variables	Research participants (*n* = 12,105)	DDH group (*n* = 207)	NDDH group (*n* = 11,898)	T/F	*P*
Birth weight (kg)	3.16 ± 0.54	3.20 ± 0.47	3.16 ± 0.54	−1.234	0.217
Month age (months)^a^				160.172	<0.001
≤1 (15–30 days)	533 (4.4)	53 (25.6)	480 (4.0)		
≤2 (31–60 days)	1,248 (10.3)	59 (28.5)	1,189 (10.0)		
≤3 (61–90 days)	2,396 (20.1)	26 (12.6)	2,422 (20.0)		
≤4 (91–120 days)	3,667 (30.8)	31 (15.0)	3,698 (30.5)		
≤5 (121–150 days)	2,418 (20.3)	24 (11.6)	2,442 (20.2)		
≤6 (151–180 days)	1,748 (14.7)	14 (6.8)	1,762 (14.6)		
Female sex				40.562	<0.001
No	5,511 (45.5)	49 (23.7)	5,462 (45.9)		
Yes	6,594 (54.5)	158 (76.3)	6,436 (54.1)		
First-degree family history of DDH				58.591	<0.001
No	12,021 (99.3)	196 (94.7)	11,825 (99.4)		
Yes	84 (0.7)	11 (5.3)	73 (0.6)		
Low-birth-weight infant (<2,500 g)				1.394	0.238
No	10,937 (90.4)	192 (92.8)	10,745 (90.3)		
Yes	1,168 (9.6)	15 (7.2)	1,153 (9.7)		
Macrosomia (≥4,000 g)				0.950	0.330
No	11,621 (96.0)	196 (94.7)	11,425 (96.0)		
Yes	484 (4.0)	11 (5.3)	473 (4.0)		
Born in winter				0.216	0.642
No	8,712 (72.0)	146 (70.5)	8,566 (72.0)		
Yes	3,393 (28.0)	61 (29.5)	3,332 (28.0)		
Premature birth				3.279	0.070
No	10,917 (90.2)	179 (86.5)	10,738 (90.3)		
Yes	1,188 (9.8)	28 (13.5)	1,160 (9.7)		
Cesarean section				0.128	0.721
No	6,987 (57.7)	122 (58.9)	6,865 (57.7)		
Yes	5,118 (42.3)	85 (41.1)	5,033 (42.3)		
Multiple births				0.029	0.864
No	11,664 (96.4)	199 (96.1)	11,465 (96.4)		
Yes	441 (3.6)	8 (3.9)	433 (3.6)		
Firstborn child				12.373	<0.001
No	4,705 (38.9)	56 (27.1)	4,649 (39.1)		
Yes	7,400 (61.1)	151 (72.9)	7,249 (60.9)		
Breech at presentation				70.234	<0.001
No	10,880 (89.9)	150 (72.5)	10,730 (90.2)		
Yes	1,225 (10.1)	57 (27.5)	1,168 (9.8)		
Oligohydramnios				63.509	<0.001
No	11,864 (98.0)	187 (90.3)	11,677 (98.1)		
Yes	241 (2.0)	20 (9.7)	221 (1.9)		
Swaddling				40.146	<0.001
o	9,486 (78.4)	125 (60.4)	9,361 (78.7)		
Yes	2,619 (21.6)	82 (39.6)	2,537 (21.3)		
Frog leg position or vertical hold				29.040	<0.001
No	4,709 (38.9)	118 (57.0)	4,591 (38.6)		
Yes	7,396 (61.1)	89 (43.0)	7,307 (61.4)		
Skin pattern asymmetry				38.610	<0.001
No	6,783 (56.0)	72 (34.8)	6,711 (56.4)		
Yes	5,322 (44.0)	135 (65.2)	5,187 (43.6)		
Restriction of hip abduction				205.377	<0.001
No	11,160 (92.2)	136 (65.7)	11,024 (92.7)		
Yes	945 (7.8)	71 (34.3)	874 (7.3)		
Leg length discrepancy				291.069	<0.001
No	11,888 (98.2)	171 (82.6)	11,717 (98.5)		
Yes	217 (1.8)	36 (17.4)	181 (1.5)		
Combined malformation				114.732	<0.001
No	11,604 (95.9)	168 (81.2)	11,436 (96.1)		
Yes	501 (4.1)	39 (18.8)	462 (3.9)		

DDH, developmental dysplasia of the hip.

a Continuous variables are expressed as mean ± standard deviation; categorical variables are expressed as number of cases (percentage). An independent sample t-test was used for continuous variables and the Chi-squared test for categorical variables. Statistical values are expressed as T-values for continuous variables and F-values for categorical variables. First-degree family history of DDH, oligohydramnios, and leg length discrepancy were corrected using a continuity correction.

## 3 Results

### 3.1 Baseline characteristics of the study participants

The training and test sets included a total of 12,105 participants, including 207 patients in the DDH group and 11,898 patients in the non-DDH (NDDH) group (Table 1). Among the patients in the DDH group, 22 patients were classified as having Graf type III and 6 patients as having Graf type IV. Basic information included birth weight and age (in months). Birth weight was normally distributed (*P* > 0.05). Seventeen variables were included based on epidemiologic information. The difference in prevalence among the age groups was statistically significant (*P* < 0.05), and the prevalence showed a decreasing trend with the increase in age (*P* < 0.05). In the DDH group, the prevalence of female sex, first-degree family history of DDH, being the firstborn child, breech at presentation, oligohydramnios, swaddling, skin pattern asymmetry, restriction of hip abduction, leg length discrepancy, and combined malformation were all higher than those in the NDDH group, and the difference was statistically significant (*P* < 0.05). In addition, the prevalence of being in a frog leg position or vertical holding was higher in the NDDH group than in the DDH group, and the difference was statistically significant (*P* < 0.05). There were no statistically significant differences in distribution between the two groups (*P* > 0.05) in terms of low birth weight, macrosomia, birth in winter, premature birth, cesarean section, and multiple births, as shown in Table 1.

### 3.2 Risk factor analysis

In the univariate analysis, we found that 11 variables had *P*-values <0.001, with a *P*-value of 0.07 for premature birth, and all other variables were >0.2. In conjunction with the epidemiology of DDH, we made an attempt to retain premature birth, and therefore, we used *P* > 0.1 as a criterion for excluding variables. Variables with *P* > 0.1 were excluded (i.e., birth weight, low-birth-weight infant, macrosomia, birth in winter, cesarean section, and multiple births).

Age (in months) was treated as an independent covariate, and a multivariate logistic regression analysis retained 12 significant risk factors (*P* < 0.1) for model building, as shown in Table 2.

**Table 2:** A multivariate logistic regression analysis of variables related to DDH.

Variables	β	OR	95% CI	*P*-value
Month age (months)				
≤1 (15–30 days)	Reference			
≤2 (31–60 days)	−0.69	0.502	0.318–0.792	0.003
≤3 (61–90 days)	−2.13	0.119	0.069–0.206	<0.001
≤4 (91–120 days)	−2.313	0.099	0.058–0.168	<0.001
≤5 (121–150 days)	−2.304	0.100	0.057–0.176	<0.001
≤6 (151–180 days)	−2.341	0.096	0.050–0.187	<0.001
Female sex				
No	Reference			
Yes	1.008	2.739	1.914–3.920	<0.001
First-degree family history of DDH				
No	Reference			
Yes	3.736	41.916	18.614–94.386	<0.001
Premature birth				
No	Reference			
Yes	0.691	1.997	1.266–3.148	0.003
Firstborn child				
No	Reference			
Yes	0.514	1.672	1.183–2.365	0.004
Breech at presentation				
No	Reference			
Yes	0.991	2.695	1.855–3.914	<0.001
Oligohydramnios				
No	Reference			
Yes	1.694	5.441	2.919–10.144	<0.001
Skin pattern asymmetry				
No	Reference			
Yes	1.696	5.452	3.829–7.761	<0.001
Restriction of hip abduction				
No	Reference			
Yes	2.365	10.649	7.282–15.572	<0.001
Leg length discrepancy				
No	Reference			
Yes	2.406	11.093	6.535–18.831	<0.001
Combined malformation				
No	Reference			
Yes	1.369	3.931	2.483–6.222	<0.001
Swaddling				
No	Reference			
Yes	0.973	2.646	1.892–3.702	<0.001
Frog leg position or vertical hold				
No	Reference			
Yes	−0.741	0.476	0.341–0.666	<0.001

OR, odds ratio; 95% CI, 95% CI for the odds ratio; β, regression coefficient.

*P* < 0.05 represents a statistically significant difference.

### 3.3 Model performance

Among the models trained on 1:2 data, the SVC model displayed the best overall performance, with the best metrics, except for the AUC, where it was second only to the RF and LR models, as shown in Table 3. The ROC curves for the 1:2 data models are shown in Figure 2. For the models trained on 1:1 data, the XGBoost model displayed the best overall performance, with the best metrics, except for specificity, where it was second only to the KNN model, as shown in Table 4. The ROC curves for the 1:1 data models are shown in Figure 3.

**Table 3:** Performance of 1:2 data models (207 cases in the DDH group and 414 cases in the NDDH group).

Models	AUC (95% CI)	Accuracy	Precision	F1 score	Recall (sensitivity)	Specificity
RF	0.923^a^ (0.881–0.956)	0.840	0.722	0.722	0.722	0.888
SVC	0.894 (0.842–0.942)	0.864^a^	0.771^a^	0.761^a^	0.750^a^	0.910^a^
LR	0.900 (0.851–0.945)	0.856	0.750	0.750	0.750^a^	0.899
KNN	0.897 (0.847–0.942)	0.800	0.667	0.638	0.611	0.876
LightGBM	0.881 (0.815–0.937)	0.832	0.742	0.687	0.639	0.910^a^
XGBoost	0.887 (0.826–0.937)	0.840	0.750	0.700	0.667	0.910^a^

AUC, area under the receiver operating characteristic curve; RF, random forest classification model; SVC, support vector machine classification model; LR, logistic regression model; KNN, k-nearest neighbor classification model; LightGBM, light gradient boosting machine classification model; XGBoost, extreme gradient boosting classification model.

a The highest value of each item.

**Figure 2:**
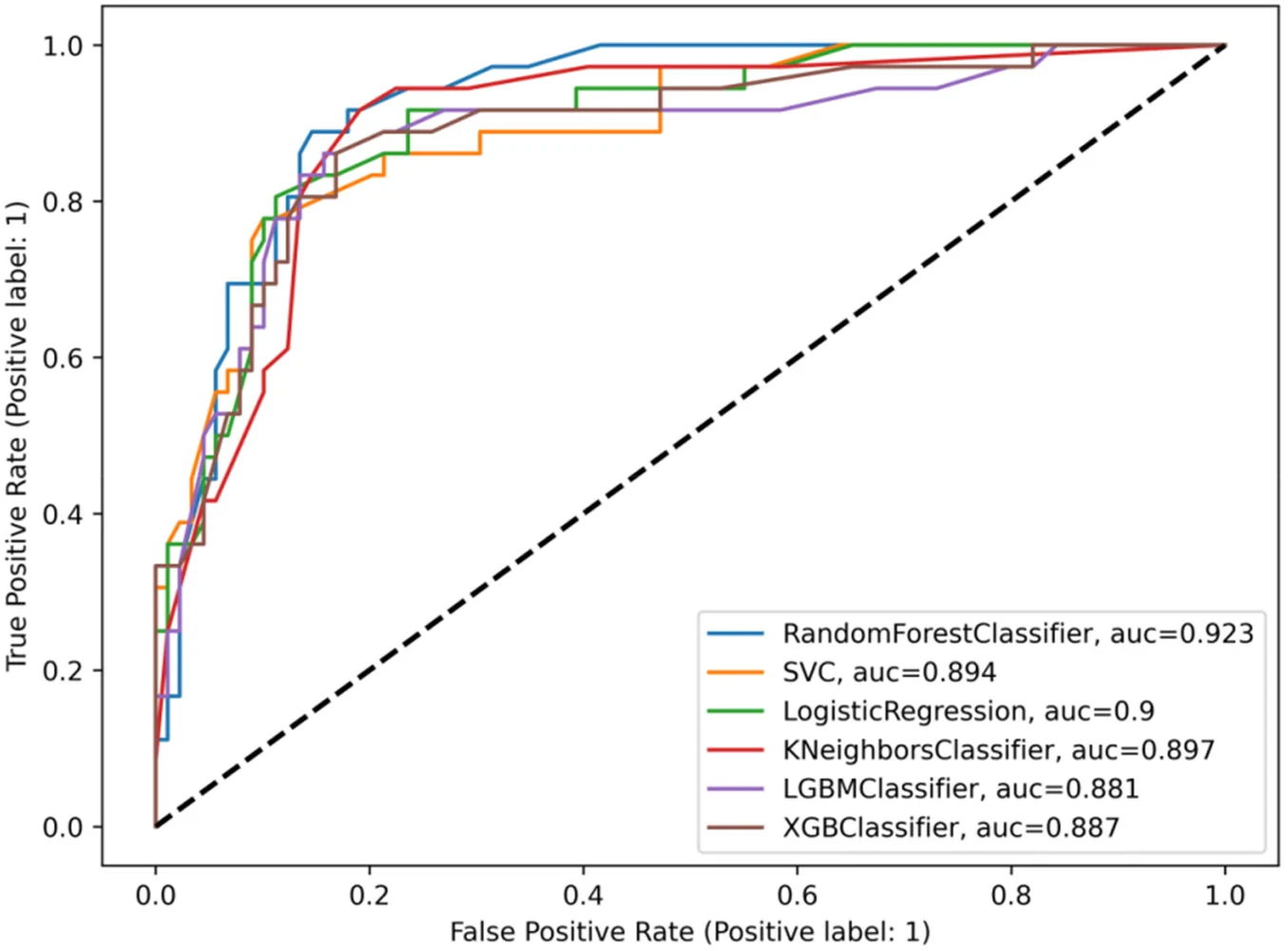
ROC curves for 1:2 data models. AUC, area under the receiver operating characteristic curve; RandomForestClassifier, random forest classification model; SVC, support vector machine classification model; Logistic Regression, logistic regression model; KNeighborsClassifier, k-nearest neighbor classification model; LGBMClassifier, light gradient boosting machine classification model; XGBClassifier, extreme gradient boosting classification model.

**Table 4:** Performance of 1:1 data models (207 cases in the DDH group and 207 cases in the NDDH group).

Models	AUC (95% CI)	Accuracy	Precision	F1 score	Recall (sensitivity)	Specificity
RF	0.844 (0.765–0.909)	0.747	0.700	0.727	0.757	0.739
SVC	0.861 (0.791–0.920)	0.759	0.689	0.756	0.838	0.696
LR	0.876 (0.804–0.939)	0.783	0.732	0.769	0.811	0.761
KNN	0.858 (0.793–0.920)	0.771	0.765	0.732	0.703	0.826^a^
LightGBM	0.857 (0.785–0.925)	0.783	0.732	0.769	0.811	0.761
XGBoost	0.883^a^ (0.815–0.944)	0.807^a^	0.744^a^	0.800^a^	0.865^a^	0.761

AUC, area under the receiver operating characteristic curve; RF, random forest classification model; SVC, support vector machine classification model; LR, logistic regression model; KNN, k-nearest neighbor classification model; LightGBM, light gradient boosting machine classification model; XGBoost, extreme gradient boosting classification model.

a The highest value of each item.

**Figure 3:**
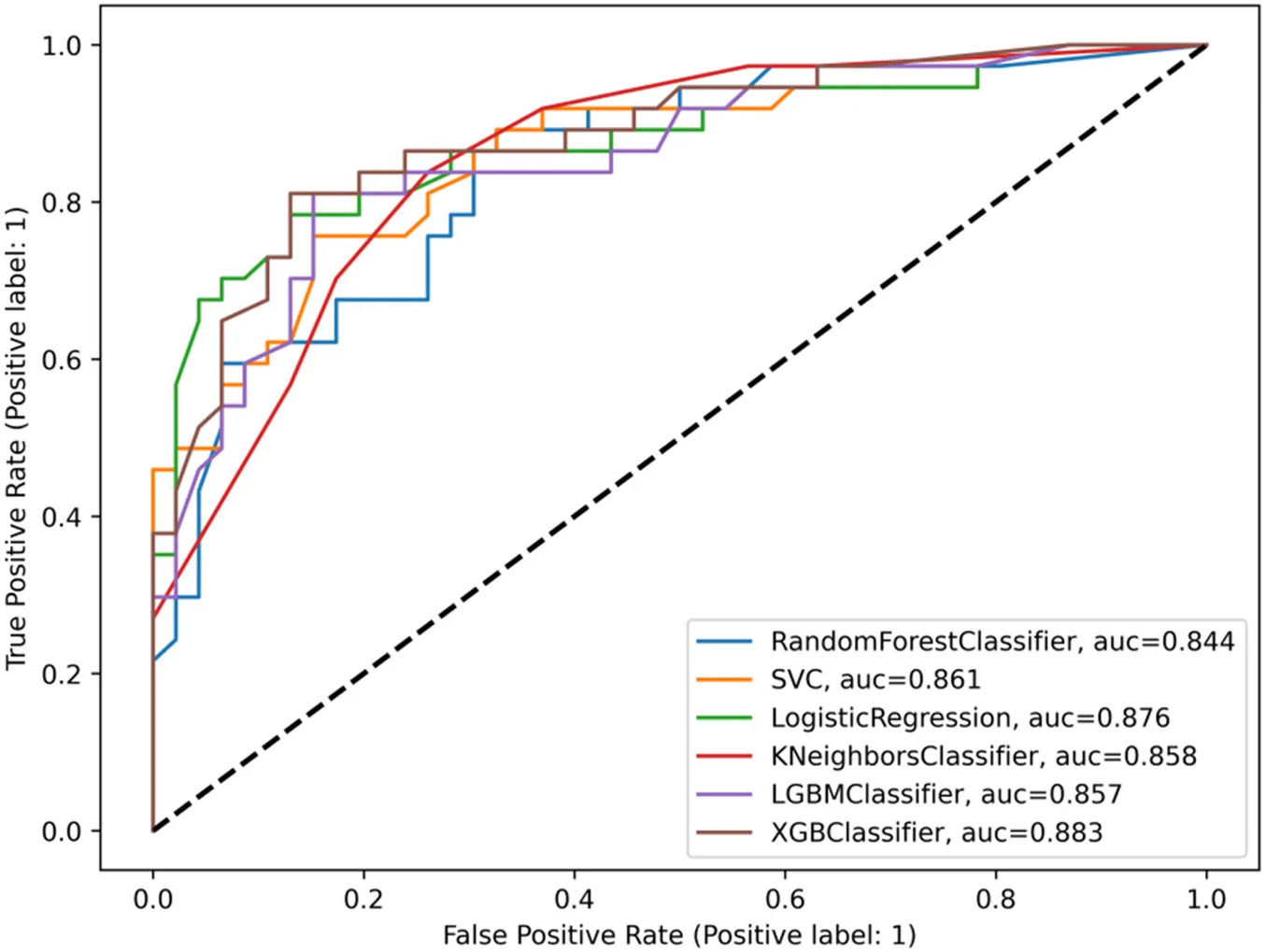
ROC curves for 1:1 data models. AUC, area under the receiver operating characteristic curve; RandomForestClassifier, random forest classification model; SVC, support vector machine classification model; Logistic Regression, logistic regression model; KNeighborsClassifier, k-nearest neighbor classification model; LGBMClassifier, light gradient boosting machine classification model; XGBClassifier, extreme gradient boosting classification model.

Comparing the models constructed from the two proportions of data, it was found that the models constructed from 1:1 data had higher F1 scores and recall (sensitivity) than those built from 1:2 data. On the other hand, the models constructed from 1:2 data had higher AUC, precision, accuracy, and specificity than those built from 1:1 data.

### 3.4 External validation

The model was externally validated for a total of 80 patients; among them, there were 40 positive and 40 negative cases each. We further externally validated the XGBoost model, which was trained on 1:1 data, and the results are as follows: area under the curve: 0.909, accuracy: 0.838, precision: 0.829, F1 score: 0.840, recall (sensitivity): 0.850, and specificity: 0.825. The ROC curves are shown in Figure 4.

**Figure 4:**
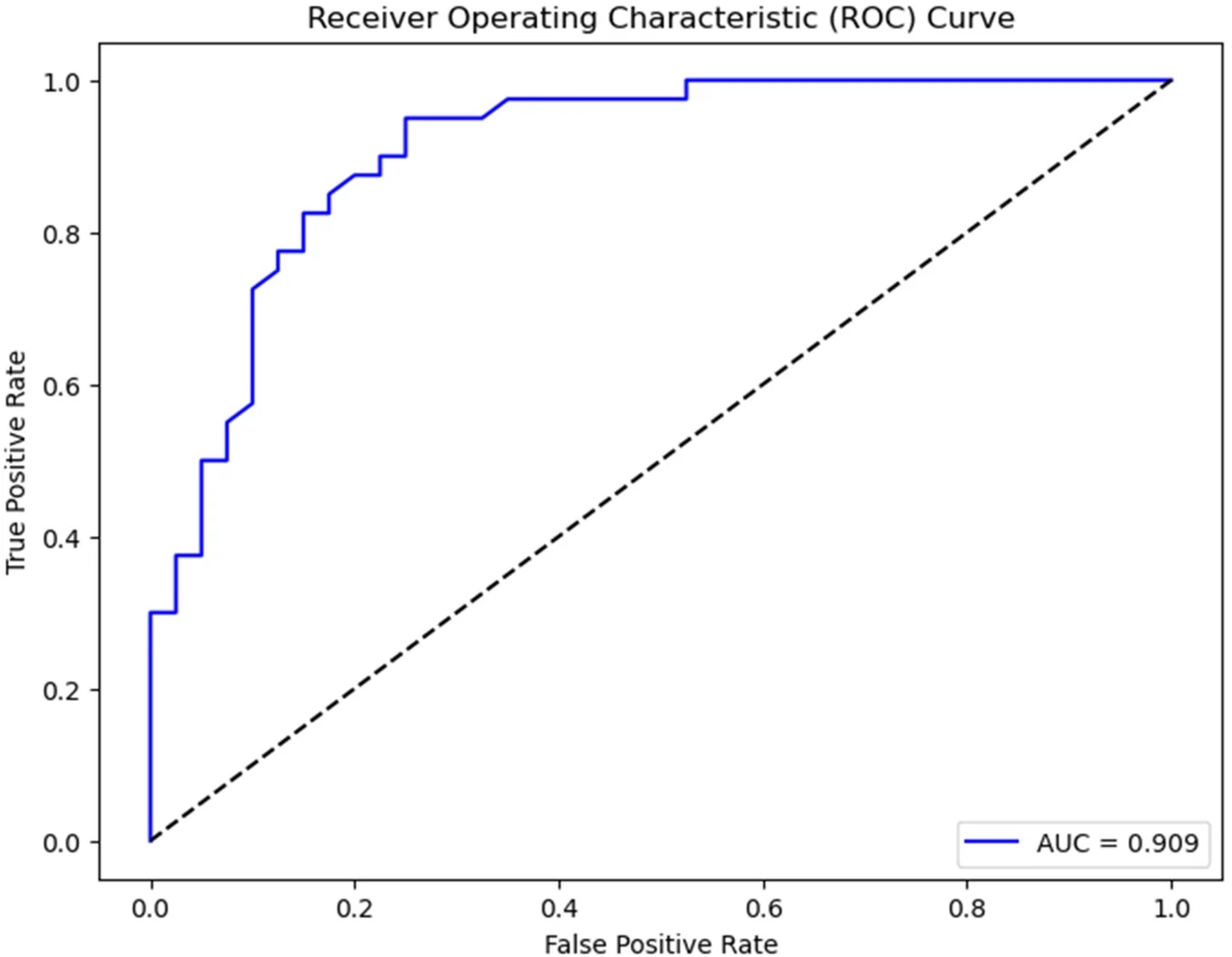
ROC curves for external validation.

### 3.5 Variable importance analysis

We further analyzed the important features in the XGBoost model trained on 1:1 data using SHAP. In this analysis, the higher the mean of the absolute SHAP values of the 12 variables, the greater the influence of the variables on the model output (13) (Figure 5). Furthermore, we observed that skin pattern asymmetry, restriction of hip abduction, swaddling, breech at presentation, and female sex were the five top most important variables for DDH prediction using the XGBoost model. The SHAP summary plot displayed in Figure 6 shows the effect of the feature variables on the predicted probability. We found that restriction of hip abduction, skin pattern asymmetry, breech at presentation, swaddling, being the firstborn child, female sex, combined malformation, leg length discrepancy, no frog leg position or vertical holding, oligohydramnios, first-degree family history of DDH, and premature birth were all associated with an increased risk of developing DDH.

**Figure 5:**
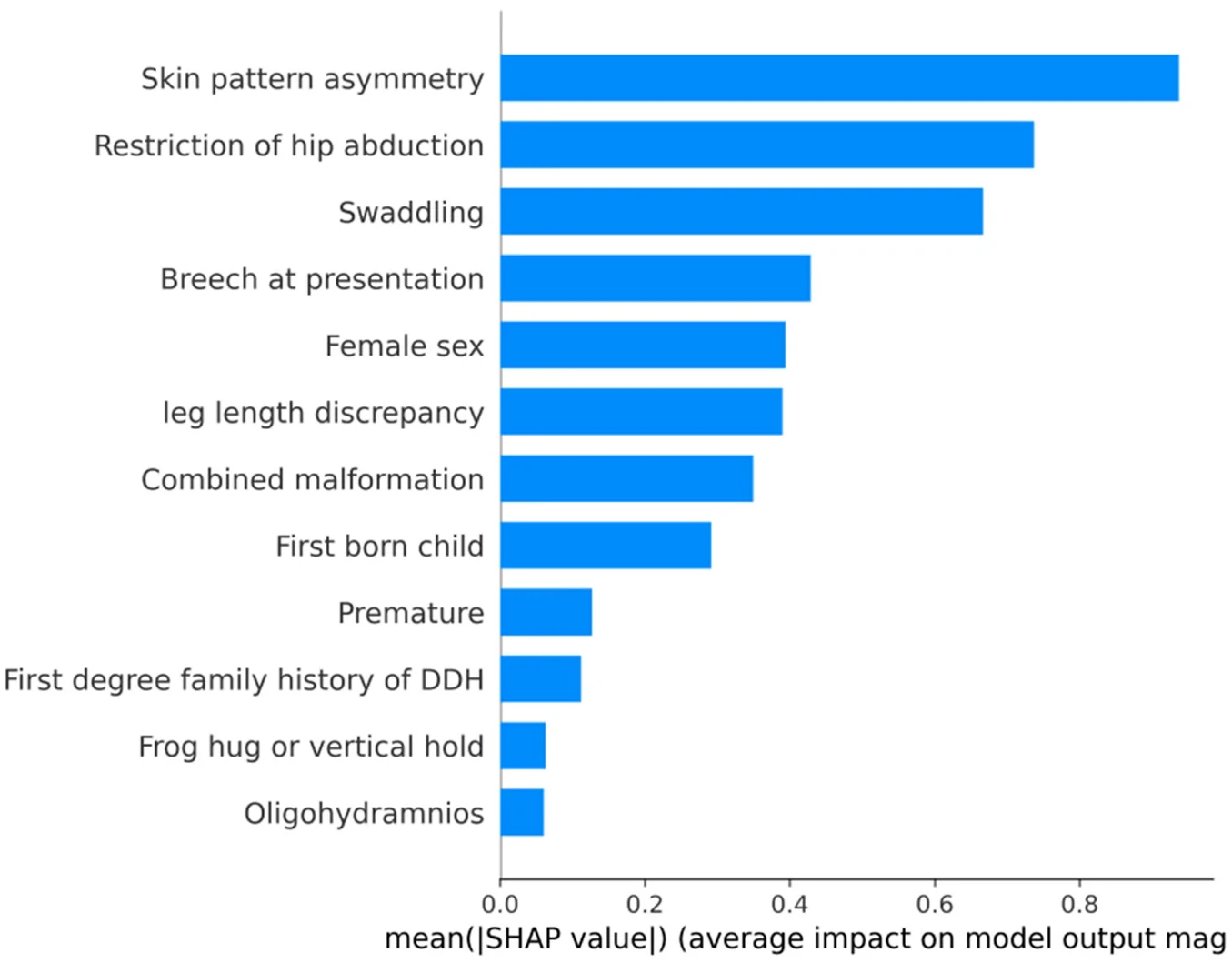
Features of importance derived from the XGBoost model. The SHAP value represents the high importance of the feature. The higher the SHAP value of a feature, the more probable the onset of DDH.

**Figure 6:**
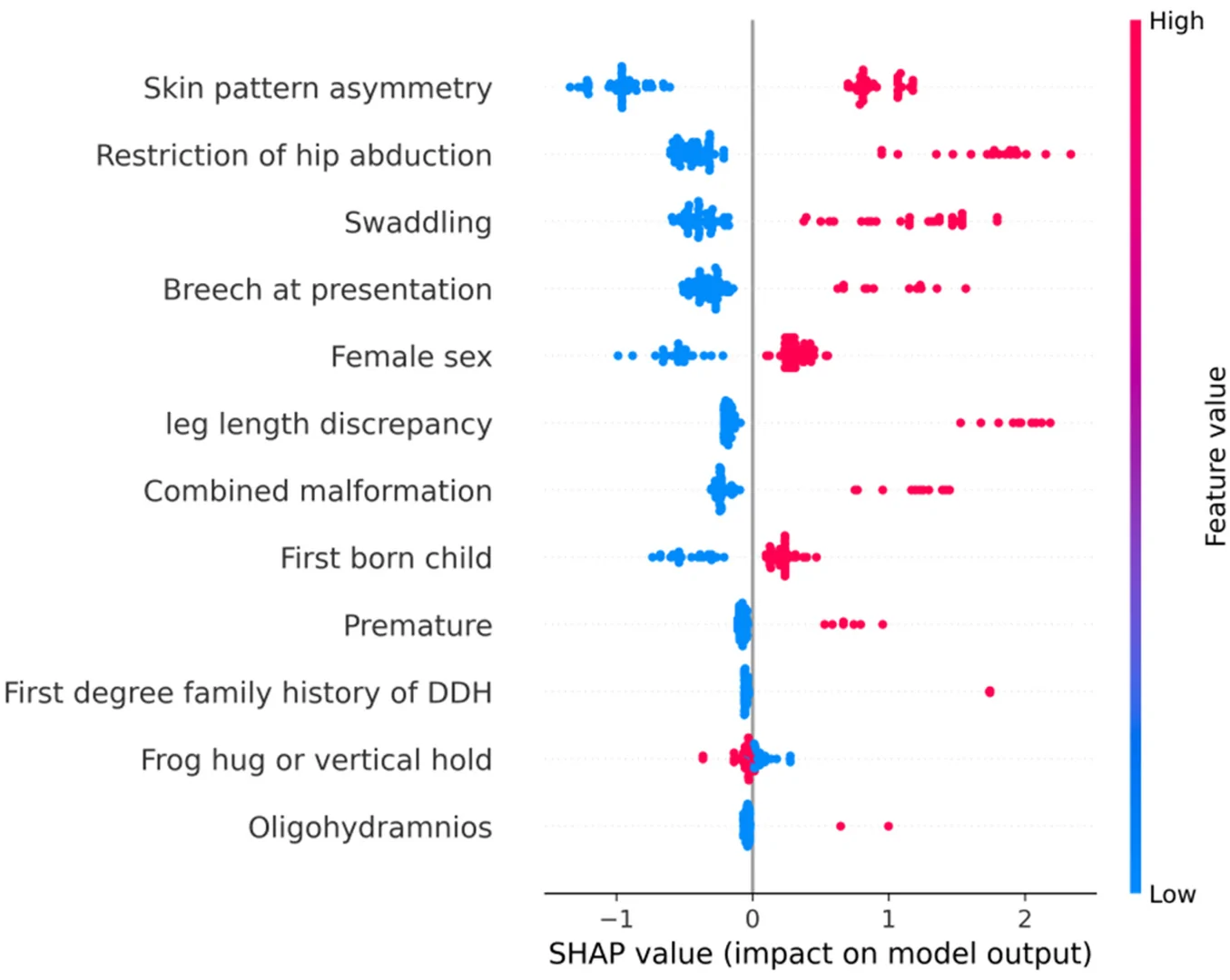
A SHAP summary graph of the XGBoost model. There are 12 variables, and the colors represent the eigenvalues of the variables, with eigenvalues in red higher than those in blue. Vertical accumulation depicts density.

## 4 Discussion

DDH is characterized by insufficient acetabular coverage of the femoral head. Without early treatment, it can progress to abnormal hip mechanics and eventual adult arthroplasty (14, 15). Because of the subjective dependence on clinical hip screening and the high rate of false positives and false negatives, hip ultrasonography based on Graf's method is currently the most accurate diagnostic tool for early DDH detection (16). However, hip screening protocols vary widely and are debated in different countries and regions (17, 18). Currently, different screening strategies are used in different countries and regions, which mainly comprise universal or selective ultrasound screening. Universal ultrasound screening can reduce the late detection rate (19); however, it carries a risk of overtreatment (4). In addition, the ultrasound procedure is cumbersome and requires a high level of expertise. Furthermore, the scope of selective ultrasound screening is difficult to determine, and a small screening range poses a risk for delayed treatment for the child (20). As such, screening for risk factors of DDH and establishing individualized risk-stratification models are necessary to help clinicians perform the initial screening of infants and children and to decide whether hip ultrasonography is required.

An important methodological decision concerned the handling of class imbalance. The raw cohort was highly imbalanced (1.7% positive), and age-stratified undersampling inevitably discarded part of the information of patients without DDH. However, this approach was chosen because the prevalence of DDH declined markedly with the increase in age; matching the age distribution of the negative and positive groups prevented age from dominating the models and allowed learning of the risk-factor associations.

Twelve variables were screened for use in constructing the model through a univariate analysis and a multifactorial logistic regression analysis of 18 possible risk factors. Previous studies have shown that genetic factors, hormone levels, and mechanical factors have been suggested as possible influences in the development of DDH. Female sex has a much higher prevalence than male sex, which may be related to ligamentous laxity in female newborns due to maternal estrogen (21). According to the theory of mechanics, breech at presentation, swaddling, no frog leg position or vertical holding, and oligohydramnios are risk factors for DDH (22). Restriction of hip abduction, skin pattern asymmetry, and leg length discrepancy are all common clinical manifestations of DDH (23). Other important risk factors include oblique neck and being the firstborn child (24, 25), which are consistent with our findings.

Prediction models can assist with early detection for potential patients with DDH. A growing number of artificial intelligence (AI) studies for DDH have focused on deep learning–based hip ultrasonography or pelvic radiography for automated diagnosis (26); these approaches operate at the diagnostic stage and require imaging equipment and trained operators. In contrast, relatively few studies have applied machine learning to demographic risk factors and physical examination findings for preimaging screening of DDH. Consistent with previous conventional risk-stratification tools—described by Pulik et al. (27), who developed a logistic regression risk assessment tool (AUC: 0.81) without external validation, and Roposch et al. (28), whose model reached a C-statistic of 0.9—our XGBoost model achieved an AUC of 0.883 in internal testing and of 0.909 in external validation. Moreover, unlike the risk-factor counting rule proposed by Ionescu et al. (29) (referral for ultrasonography when two or more risk factors or clinical signs are present), our model provides a continuous, individualized risk estimate that integrates both risk factors and clinical signs, potentially enabling risk-based prioritization when ultrasound resources are limited. Therefore, our model is positioned at the preimaging screening and risk-stratification stage and is complementary to imaging-based AI approaches. Prospective, multicenter studies are warranted to confirm its clinical utility.

In this study, 12 independent risk factors were selected for modeling after five variables were eliminated by univariate and multifactorial logistic regression analyses. Overall, the classification performance of the 12 constructed models was satisfactory, and the models could be used to identify infants at high risk for DDH for referral to hip ultrasonography. We discovered that the models trained on 1:2 data with recall (sensitivity) (maximum: 0.75) performed poorer than those trained on 1:1 data (maximum: 0.865). However, the specificity (maximum: 0.910) of the 1:2 data was generally higher than that of the 1:1 data (maximum: 0.826) because the negative data in the 1:2 data were almost twice those in the positive data, which introduced more bias toward predicting a negative outcome in the models. Owing to the serious consequences of DDH misdiagnosis, we prefer models with high recall (sensitivity). In addition, the models trained on the 1:1 data had a higher F1 score than those trained on the 1:2 data; therefore, we believe that the XGBoost [AUC: 0.883, accuracy: 0.807, precision: 0.744, F1 score: 0.800, recall (sensitivity): 0.865, and specificity: 0.761] model trained on the 1:1 data is the best model. The model has been externally validated in other hospitals with a slight increase in all metrics, except for a slight decrease in recall (sensitivity; 0.850). We propose that this model can help clinicians identify infants at high risk for DDH and triage them for hip ultrasonography, thereby supporting DDH screening. The XGBoost model is an efficient gradient boosting decision tree algorithm (30) that has the advantages of high accuracy, robustness, speed, and maximum predictive performance and can be widely used for predictive model building and risk identification in the medical field (31, 32). Notably, it is difficult to visualize the algorithm and interpret the model's output (33). Therefore, we introduced SHAP to demonstrate the output of the best model to help clinicians understand the mechanisms of the model's output.

According to the SHAP analysis of the XGBoost model, the two physical examination findings—restriction of hip abduction and skin pattern asymmetry—were the most important factors, which suggests that extra attention should be paid to physical examination during the screening of DDH. Among the epidemiologic risk factors, breech at presentation and being the firstborn child were also among the top five most important variables in the model. Avoidance of swaddling among holding practices may reduce the risk of DDH, which is the fourth most important variable in the model output, because of the fact that swaddling can keep the hip in extension for a long period of time, which is detrimental to the development of the hip joint.

This study has several limitations. (1) Selection bias may exist because our cohort included infants whose parents actively sought DDH screening. This was a single-center study, and external validation was performed only in tertiary hospitals with a relatively small sample (*n* = 80). Thus, our results provide only preliminary evidence of model transferability, and further validation covering community and rural populations across multiple centers is required. (2) The number of patients positive for DDH was limited (*n* = 207), which restricted feature space and model complexity. Patients with complex cases of hip dislocation (syndromic, pathological, paralytic, and teratoid) were excluded to ensure cohort homogeneity, and hence, the model cannot be generalized to these subpopulations. (3) As a retrospective analysis, causal inference cannot be drawn, and unmeasured confounders may remain. (4) Additional DDH-related risk factors such as altitude and late-gestational breech positioning can be incorporated in future models (34, 35). (5) Variable prescreening using univariate and multivariate logistic regression was carried out on the full source cohort before train-test splitting, which may cause subtle information leakage. Nested cross-validation with fold-specific feature selection would be methodologically superior but was not feasible in this study, given the limited DDH-positive sample size. (6) Although age-stratified undersampling effectively mitigated age confounding, class-weighted learning and oversampling approaches were not implemented in the current work. Future work may compare different imbalance-adjustment strategies for DDH risk prediction.

In addition, calibration metrics (calibration curve, Brier score) and decision-curve analysis were not performed in the current study. The internal test datasets were artificially balanced (1:1 or 1:2 case–control ratio) after age-stratified undersampling and do not reflect the real-world DDH prevalence of 1.7%. Similarly, the external validation dataset was artificially balanced. Calibration and decision-curve analysis require data with natural disease prevalence in a population to generate valid absolute risk estimates and net-benefit metrics. Probabilities derived from artificially balanced cohorts cannot represent absolute individual risk in routine clinical practice. Therefore, our model should be regarded as a relative-risk ranking tool to prioritize infants for hip ultrasound referral rather than as a tool that provides well-calibrated absolute risk probabilities. Future prospective cohorts with natural DDH prevalence are needed to formally evaluate model calibration and clinical utility.

## Data Availability

The raw data supporting the conclusions of this article will be made available by the authors, without undue reservation.
