# Supplementary material for: Machine learning–based personalized prediction of developmental dysplasia of the hip using risk factors and clinical examination
Source: Front Digit Health. 2026 Oct 14;8:1912106. doi: 10.3389/fdgth.2026.1912106 (PMC13713713; doi:10.3389/fdgth.2026.1912106)
Supplement: Supplementary file 1 [file Table1.pdf]

Supplementary Table S1. Hyperparameter search grids and optimal parameters for the six machine learning algorithms in the 1:1 and 1:2 cohorts.

Algorithm	Grid searched	Optimal parameters (1:1 cohort)	Optimal parameters (1:2 cohort)	Random seed
Random Forest	n_estimators: [100, 200, 300]; criterion: [gini, entropy]; max_features: [sqrt]	criterion='entropy'; n_estimators=300; max_features='sqrt'	criterion='gini'; n_estimators=300; max_features='sqrt'	random_state=42
SVC	kernel: [linear, rbf]; C: [1.0, 1.5]; gamma: [auto]	kernel='linear'; C=1.5; gamma='auto'	kernel='linear'; C=1.0; gamma='auto'	random_state=42
Logistic Regression	tol: [1e-4, 1e-5]; max_iter: [100, 200, 300]; solver: [liblinear]	solver='liblinear'; tol=1e-4; max_iter=100; C=1.0	Same as 1:1	random_state=42
KNN	n_neighbors: 1–29; weights: [distance, uniform]; p: [1, 2]	n_neighbors=8; weights='uniform'; p=1	n_neighbors=17; weights='uniform'; p=2	Not applicable
LightGBM	learning_rate: [0.1, 0.01]; max_depth: [3, 5, 7]; n_estimators: [100, 200]	learning_rate=0.1; max_depth=3; n_estimators=100	Same as 1:1	random_state=42
XGBoost *	learning_rate: [0.1, 0.01]; max_depth: [3, 5, 7]; n_estimators: [100, 200]	learning_rate=0.1; max_depth=3; n_estimators=100; objective='binary:logistic'	Same as 1:1	random_state=42

*Note: Hyperparameters were optimized by grid search with 3-fold cross-validation (GridSearchCV, cv=3) using the area under the ROC curve (scoring='roc_auc') as the optimization criterion. The optimal parameters are shown for the models trained on the 1:1 and 1:2 balanced cohorts, respectively. *XGBoost trained on the 1:1 cohort was the best-performing model and was selected for the subsequent model interpretation (SHAP) and external validation analyses. KNN contains no random components; therefore, no random seed is required.*
